# Exogenous IFN-γ *ex vivo* shapes the alloreactive T-cell repertoire by inhibition of Th17 responses and generation of functional Foxp3^+^ regulatory T cells

**DOI:** 10.1002/eji.200838411

**Published:** 2008-09

**Authors:** Gang Feng, Wenda Gao, Terry B Strom, Mohamed Oukka, Ross S Francis, Kathryn J Wood, Andrew Bushell

**Affiliations:** 1 Transplantation Research Immunology Group, Nuffield Department of Surgery, University of Oxford, John Radcliffe HospitalOxford, UK; 2 Transplant Research Center, Beth Israel Deaconess Medical Center, Harvard Medical SchoolBoston, MA, USA; 3 Center for Neurologic Diseases, Brigham and Women's Hospital, Harvard Medical SchoolBoston, MA, USA

**Keywords:** Cellular therapy, IFN-γ, Regulatory T cells, Transplant rejection

## Abstract

Interferon (IFN)-γ was originally characterized as a pro-inflammatory cytokine with T helper type 1-inducing activity, but subsequent work has demonstrated that mice deficient in IFN-γ or IFN-γ receptor show exacerbated inflammatory responses and accelerated allograft rejection, suggesting that IFN-γ also has important immunoregulatory functions. Here, we demonstrate that *ex vivo* IFN-γ conditioning of CD4 T cells driven by allogeneic immature dendritic cells (DC) results in the emergence of a Foxp3^+^ regulatory T-cell (Treg)- dominant population that can prevent allograft rejection. The development of this population involves conversion of non-Treg precursors, preferential induction of activation-induced cell death within the non-Treg population and suppression of Th2 and Th17 responses. The suppressive activity of IFN-γ is dependent on the transcription factor signal transducer and activator of transcription 1 and is mediated by induced nitric oxide. These data indicate not only how IFN-γ could be used to shape beneficial immune responses *ex vivo* for possible cell therapy but also provide some mechanistic insights that may be relevant to exacerbated inflammatory responses noted in several autoimmune and transplant models with IFN-γ deficiency.

## Introduction

The role of interferon (IFN)-γ in cellular immunity is somewhat paradoxical in that, although it is usually considered to be a pro-inflammatory effector cytokine, increasing evidence suggests that it plays a non-redundant immunoregulatory role. For example, experimental autoimmune encephalomyelitis (EAE) and collagen-induced arthritis (CIA) have been historically associated with IFN-γ-producing Th1-dominant responses 1, but mice deficient in IFN-γ or IFN-γ receptor develop EAE at an accelerated rate 2–4 and, similarly, deficiency in IFN-γ or IFN-γ receptor leads to more severe CIA and the development of CIA in otherwise non-susceptible strains 5–7. IFN-γ can also have immunomodulatory effects on antigen-presenting cells and a recent report has demonstrated that adoptive transfer of IFN-γ-stimulated monocyte-derived cells promotes the resolution of experimental colitis and is associated with an enrichment of CD25^+^ Foxp3^+^ T cells 8.

A paradoxical role for IFN-γ is also seen in organ transplantation 9–11. Allograft rejection is a process frequently associated with a dominant Th1 IFN-γ response whereas the absence of intragraft IFN-γ often correlates with long-term graft survival 12,13. However, IFN-γ appears not to be essential for acute cellular rejection as both IFN-γ-deficient and wild-type mice reject cardiac allografts with similar kinetics 14,15 and at least one study has demonstrated that IFN-γ^−/−^ recipients reject skin allografts more rapidly than their wild-type littermates 9. In fact, IFN-γ may be required for successful engraftment 9,16,17.

Although the classical view of IFN-γ is that it favors Th1 cell development 18,19, IFN-γ also has regulatory functions. For example, IFN-γ can inhibit the proliferation of IL-4-producing Th2 cells 20 and suppress the development of Th17 effector cells now known to play an important role in many autoimmune models 21,22. In addition, IFN-γ also plays an important role in maintenance of T-cell homeostasis by inducing apoptosis-dependent activation-induced cell death (AICD) to limit T-cell expansion following antigen encounter 23–28. In the context of adaptive regulation, we have recently shown that IFN-γ is produced rapidly and transiently by alloantigen-reactive Treg following reactivation and that this is required for their functional activity *in vivo* 29. IFN-γ can induce indoleamine 2,3-dioxygenase in several cell types and this enzyme has been shown to play an important role in limiting T-cell responses *in vivo* 30–32.

In this study, we demonstrate that *ex vivo* exposure of CD4^+^T cells to allogeneic bone marrow-derived dendritic cells (DC) in the presence of IFN-γ results in a Treg population that prevents allograft rejection without further manipulation. The data indicate that IFN-γ shifts the balance of the T-cell population in favor of an enhanced proportion of Foxp3^+^ Treg by selectively enhancing cell death in the non-Treg population and by promoting direct conversion of non-Treg precursors. Experiments using DC from IFN-γ receptor-deficient mice demonstrate that this does not depend on a DC response to IFN-γ but that T-cell signalling through the signal transducer and activator of transcription (STAT)1 pathway is essential for the emergence of a dominant Treg response. Significantly, inhibition of nitric oxide synthase (NOS) abolishes the emergence of the dominant Treg response and provision of a nitric oxide (NO) donor in the absence of IFN-γ replicates the IFN-γ effect, clearly indicating an important role for NO in this process. Overall, the data highlight a novel role of IFN-γ in the regulation of T-cell homeostasis and suggest additional possibilities for cell-based therapy in transplantation and autoimmunity.

## Results

### IFN-γ promotes the enrichment of functional Foxp3^+^ Treg

**Figure 1:**
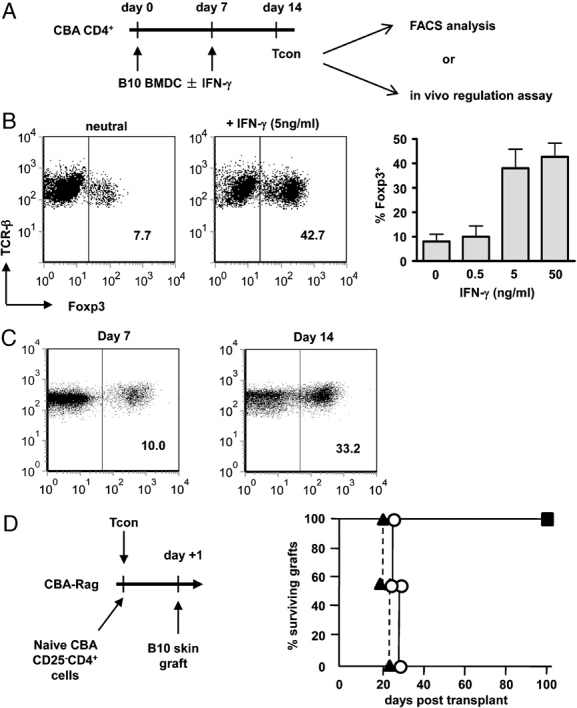
Alloreactive Foxp3^+^ T cells emerge in the presence of exogenous IFN-γ. (A) Purified naive CBA CD4^+^ T cells were co-cultured with B10 GM-CSF/TGF-β-differentiated BM DC without exogenous cytokine (neutral conditions) or in the presence of IFN-γ (5 ng/mL), restimulated under the same conditions on day 7 and harvested on day 14 for phenotypic and functional analysis. (B) Harvested populations were stained for TCR-β and Foxp3. Dot plots show representative data at 5 ng/mL IFN-γ, and the right-hand histogram shows IFN-γ dose–response data (means+SD of three independent experiments). (C) A single culture of CD4^+^ T cells driven by B10 GM-CSF/TGF-β-differentiated BM DC in the presence of IFN-γ was assayed for Foxp3 expression on day 7 after a single round of stimulation and on day 14 after restimulation on day 7. (D) Adoptive transfer protocol. All CBA-Rag^−/−^ mice were reconstituted with 1×10^5^ CD25^−^CD4^+^ cells from naive CBA mice as an effector population with or without *ex vivo* conditioned CD4 T cells (Tcon). The reconstituted mice then received a B10 skin graft the following day. Mice reconstituted with 10^5^ CD25^−^CD4^+^ cells alone acutely rejected B10 skin grafts (○; MST=22 days, *n*=4). Co-transfer of 2×10^5^ CD4 T cells conditioned with IFN-γ prevented rejection of B10 skin grafts (▪; MST>100 days, *n*=4; *p*<0.05, ▪ *versus* ○), whereas cotransfer of 2×10^5^ CD4 T cells conditioned without IFN-γ did not prevent rejection (▴; MST=19 days, *n*=4; *p*>0.05, ▴ *versus* ○). Data are representative of three independent experiments with similar group sizes.

The counter-regulation of Th2 and Th17 responses mediated by IFN-γ, together with previous observations from this laboratory indicating that Treg generation *in vivo* is impaired in the absence of IFN-γ 29, prompted us to ask whether this cytokine could be used to drive the emergence of alloreactive regulatory T cells *ex vivo*. CBA.Ca (CBA) CD4^+^ T cells were stimulated by GM-CSF/TGF-β-conditioned irradiated C57BL/10 (B10) bone marrow-derived DC (BM DC) in the presence of exogenous IFN-γ for 7 days, restimulated under the same conditions and harvested 7 days later for phenotypic and functional analysis (Fig. 1A). Under neutral conditions, the proportion of Foxp3^+^ cells remains similar to that in the input CD4^+^ population (5–10%), but the presence of IFN-γ results in a dose-dependent increase in the proportion of Foxp3^+^ cells (Fig. 1B). Replicate experiments (*n*=5) have indicated that the optimal concentration of IFN-γ in this system is 5 ng/mL, which results on average in a fivefold increase in the proportion of Foxp3^+^ cells (*p*<0.05). Unless stated otherwise, this concentration was used throughout all subsequent experiments. During development of this protocol, it became clear that, although an increase in the proportion of Foxp3^+^ cells could be seen as early as day 7, this was rather variable and generally quite low. However, at day 14, this variability was much less marked and, as shown in representative FACS plots from a single culture assayed at days 7 and 14, the proportion of Foxp3^+^ cells was significantly greater than at day 7 (Fig. 1C). Therefore, a standardized stimulation and restimulation approach was used in all experiments with cell harvest at day 14.

To determine whether these IFN-γ-conditioned CD4^+^ T cells can regulate alloreactive T-cell responses *in vivo*, naive CBA CD4^+^ T cells were co-cultured with GM-CSF/TGF-β-differentiated B10 BM DC in the presence of 5 ng/mL IFN-γ, harvested on day 14 and then adoptively transferred (2×10^5^) into CBA-Rag^−/−^ mice with CD25^−^CD4^+^ cells from naive syngeneic mice as an effector population the day before transplantation of a B10 skin graft (Fig. 1D). Reconstitution with CD25^−^CD4^+^ cells alone resulted in acute rejection [median survival time (MST) 22 days], but cotransfer of IFN-γ-conditioned cells prevented rejection, with all grafts surviving beyond 100 days (*p*<0.01). This effect was alloantigen specific in that additional cohorts of mice reconstituted with CD25^−^CD4^+^ cells and IFN-γ-conditioned cells driven by B10 BM DC rejected third-party B10.S (H2^s^) skin grafts at a rate not significantly different from those reconstituted with CD25^−^CD4^+^ cells alone (MST 17.5 days *versus* 14 days, *p*=0.13; not shown). Importantly, CD4^+^ T cells driven by GM-CSF/TGF-β B10 BM DC in the absence of IFN-γ were unable to regulate rejection in that all B10 skin allografts were rejected acutely (MST=19 days, *n*=4, *p*>0.05; Fig. 1D).

### Enrichment of Foxp3^+^ cells involves proliferation and conversion of Foxp3^–^ precursors

**Figure 2:**
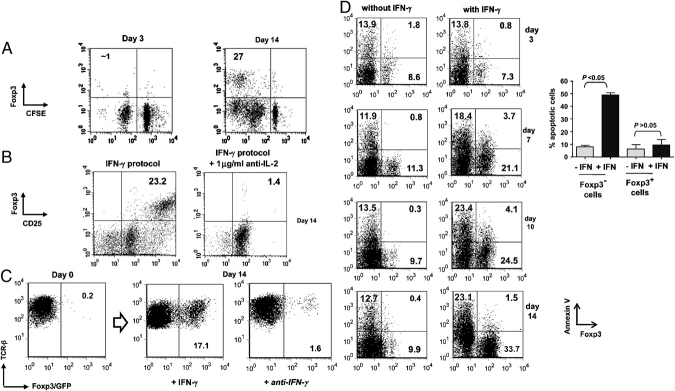
Enrichment of Foxp3^+^ cells in the IFN-γ protocol involves IL-2-dependent proliferation and conversion from Foxp3^–^ precursors. (A) CBA CD25^−^ cells were flow sorted, CFSE labeled and stimulated with B10 GM-CSF/TGF-β-differentiated BM DC in the presence of IFN-γ. Cells were harvested at day 3 to give a surrogate zero time point (CFSE intensity precluded analysis at day 0) or restimulated under the same conditions on day 7, harvested on day 14 and stained for Foxp3. (B) Purified naive CBA CD4^+^ T cells were co-cultured with B10 GM-CSF/TGF-β-differentiated BM DC in the presence of 5 ng/mL IFN-γ without or with 1 μg/mL anti-IL2 antibody (JES6-1A12), restimulated under the same conditions on day 7, harvested on day 14 and stained for CD25 and Foxp3. (C) FACS-sorted Foxp3/GFP^–^CD4^+^ T cells from naive Foxp3-GFP knock-in B6 mice were co-cultured with DBA/2 GM-CSF/TGF-β-differentiated BM DC in the presence of IFN-γ (5 ng/mL). Cells were restimulated on day 7 under the same conditions, harvested on day 14, and intracellular Foxp3/GFP expression was examined in the resultant population. In parallel, cells were stimulated in the absence of IFN-γ but the presence of anti-IFN-γ antibody. Data are representative of two independent experiments. Numbers in each dot plot indicate the frequency of cells in the quadrants shown. (D) Purified naive CBA CD4^+^ T cells were cultured with GM-CSF- and TGF-β-differentiated B10 BM DC (GT-DC) in the absence (left panel) or presence (right panel) of 5 ng/mL IFN-γ. Cells were harvested on days 3 and 7 (before restimulation on day 7) and on days 10 and 14 (after restimulation), stained for annexin V, washed, then fixed for analysis of intracellular Foxp3 expression. Numbers in each dot plot indicate the frequency of cells in each quadrant. Data are representative of four independent experiments. The histogram shows an analysis (means±SD) of four independent experiments gated on Foxp3^–^ and Foxp3^+^ cells; *p* values were calculated with the Mann–Whitney test.

The emergence of an increased proportion of Foxp3^+^ regulatory cells in this system could be explained by apoptosis of responding non-Treg cells, expansion of endogenous Foxp3^+^ cells, or conversion of non-Treg precursors since total CD4^+^ cells from naive mice were used as the input population in these experiments. To look for evidence of Treg proliferation in this system, CBA CD4^+^CD25^−^ input cells were CFSE labeled and driven with B.10 GM-CSF/TGF-β-conditioned BM DC in the presence of IFN-γ. As shown in Fig. 2A, at day 3, these cultures contained virtually no Foxp3^+^ cells (analysis at day 0 not possible due to high CFSE fluorescence intensity immediately after labeling). By day 14, there was a marked increase in the proportion of Foxp3^+^ cells and, importantly, virtually all of these were CFSE dull or negative, confirming a close correlation between Foxp3 enrichment and proliferation. Furthermore, this was dependent on endogenous IL-2 as shown by the fact that addition of neutralizing anti-IL-2 antibody practically abolished the enrichment of Foxp3^+^ cells (Fig. 2B). Since it is not possible to determine whether the enrichment shown in Fig. 2A is due to direct conversion of Foxp3^–^ cells or expansion of a small starting population of Foxp3^+^ cells contained in the sorted CD25^−^ input population, we took advantage of C57BL/6 (B6) Foxp3-GFP reporter mice 33 which allowed us to isolate GFP^−^/Foxp3^−^ cells by flow sorting. As shown in Fig. 2C, this input (day 0) population was essentially devoid of Foxp3^+^ cells, but stimulation with DBA/2 (H2^d^) GM-CSF/TGF-β-conditioned BM DC in the presence of 5 ng/mL IFN-γ resulted in significant conversion such that 17% of the cells became Foxp3^+^. The influence of IFN-γ on conversion is emphasized by the fact that when the input population was driven by GM-CSF/TGF-β-conditioned BM DC without exogenous IFN-γ but in the presence of 10 μg/mL anti-IFN-γ antibody to neutralize endogenous cytokine, conversion was reduced by tenfold. Overall, these data show that the enrichment of Foxp3^+^ cells in the IFN-γ protocol involves IL-2-dependent Treg proliferation and direct conversion of Foxp3^–^ precursors.

### IFN-γ induces cell death in alloreactive Foxp3^–^ T cells

Although the IFN-γ conditioning protocol described increases the proportion of Foxp3^+^ cells and can convert non-Treg precursors to Foxp3^+^ cells, there is no increase in the absolute number of Foxp3^+^ cells recovered at day 14 (55.3±15.5×10^3^ *versus* 38.7±18.6×10^3^ in the absence or presence of 5 ng/mL IFN-γ, *p*>0.05; not shown). In addition, the presence of IFN-γ significantly reduces the overall number of cells recovered by 2–5-fold. Given that IFN-γ contributes to T-cell homeostasis *in vivo* through inhibition of proliferation and/or by increasing AICD 23–25,28, a simple additional explanation for the increased proportion of Foxp3^+^ cells might be selective elimination of Foxp3^–^ cells and, indeed, preliminary results showed a clear correlation between proliferation and AICD in this system (not shown). To examine this in more detail, CBA CD4^+^ T cells were driven by GM-CSF/TGF-β B10 BM DC, harvested at various time points and stained for annexin V as marker of necrotic or apoptotic cell death, washed, fixed and stained for Foxp3, thus allowing analysis of apoptosis within both Foxp3^+^ and Foxp3^–^ populations. As shown in Fig. 2D, the presence of IFN-γ enhances cell death within the Foxp3^–^ population at days 7, 10 and 14, but a more comprehensive analysis at day 14 (the time of cell harvest for functional analysis; Fig. 1) showed that within the total T-cell population, the addition of IFN-γ resulted in a threefold increase in the proportion of annexin V^+^ cells. Importantly, when cells were analyzed separately on the basis of Foxp3 expression, exogenous IFN-γ had little effect on the viability of Foxp3^+^ cells (6.3±2.7 *versus* 9.5±4.3%, absence and presence of IFN-γ, respectively) but resulted in a sixfold increase in cell death within the Foxp3^–^ population (right panel, Fig. 2D). Taken together, these data show that the increased proportion of Foxp3^+^ cells and acquisition of regulatory function that occurs in this system involves both Treg conversion and elimination of potential effector cells through AICD. However, it is important to note that the ability of the resultant population to prevent allograft rejection (Fig. 1D) is not due to effector cell elimination during *ex vivo* conditioning because in the adoptive transfer model, an adequate effector population is provided in the form of exogenous naive CD25^−^ T cells. Thus, the dominant regulation shown in Fig. 1D appears to be a direct consequence of Foxp3 induction.

### Suppression of IL-6 production by DC contributes to Treg generation *ex vivo*

**Figure 3:**
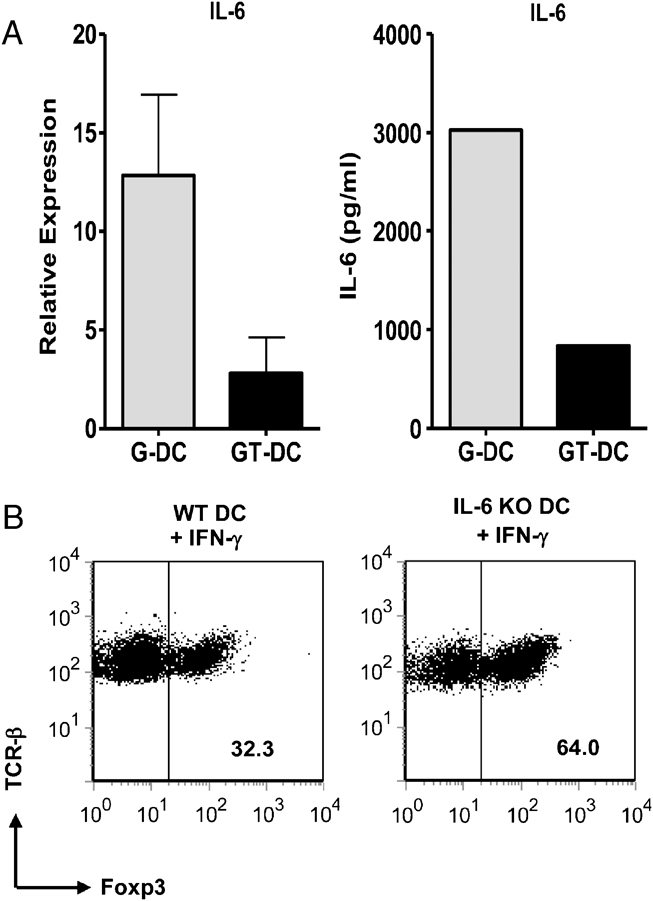
Expression of IL-6 has a negative impact on IFN-dependent Treg development. (A) BM cells were cultured in the presence of 2 ng/mL GM-CSF with (GT-DC) or without (G-DC) 2 ng/mL TGF-β, harvested at day 6 and stimulated in the absence of T cells with recombinant CD40L for 24 h to mimic reverse stimulation *via* CD40-CD40L interactions. IL-6 RT-PCR data are normalized to a housekeeping gene GAPDH, and are the means±SD of two independent experiments (left panel). Supernatants were collected for IL-6 ELISA analysis and results normalized to 4×10^5^ input cells in 2 mL medium (right panel). (B) Purified naive CBA CD4^+^ T cells were co-cultured with wild-type B6 (WT B6) or IL-6 knockout B6 (IL-6 KO B6) GM-CSF/TGF-β-differentiated BM DC in the presence of IFN-γ (5 ng/mL). Cells were restimulated on day 7 under the same conditions, harvested on day 14, and intracellular Foxp3 expression was analyzed. Numbers in each dot plot indicate the frequency of cells in the quadrant. Data are representative of two independent experiments.

The IFN-γ-conditioning system described involves two distinct phases: conditioning of BM DC with GM-CSF and TGF-β followed by stimulation of naive CD4^+^ T cells with this re-isolated DC population in the presence of IFN-γ. To understand more about the mechanisms involved in this protocol, we interrogated the APC and T-cell components of this system independently. Although mature DC are regarded as key activators of productive T-cell responses, exposure to immature DC can induce T-cell unresponsiveness 34. Phenotypic analysis of DC conditioned with GM-CSF or GM-CSF+TGF-β confirmed previous observations 35 that one effect of TGF-β is to maintain these cells in a relatively immature state as judged by reduced expression of CD40, CD80 and CD86 (not shown). In view of the fact that GM-CSF-conditioned DC produce significant amounts of IL-6, a cytokine implicated in the differentiation of Th17 cells and the negative regulation of Treg 33, we asked whether conditioning of DC with TGF-β also has an effect on IL-6 expression. BM DC were conditioned with GM-CSF only or with GM-CSF+TGF-β for 6 days, washed extensively, then stimulated with recombinant CD40L-Fc to mimic CD40-CD40L interactions without the confounding influence of other T-cell–APC interactions, and analyzed for IL-6 expression by both RT-PCR and ELISA. As shown in Fig. 3A, the addition of 2 ng/mL TGF-β as used in the DC-conditioning phase reduced both the transcription and secretion of IL-6 by approximately threefold. Since DC conditioned in the absence of TGF-β do not promote the emergence of a Foxp3-dominant response in the IFN-γ protocol (not shown), these data indicate that reduced IL-6 production is an important additional characteristic of the DC required for Treg development in this system. Furthermore, when GM-CSF-TGF-β-conditioned DC obtained from IL-6-deficient mice were used to stimulate CBA CD4^+^ T cells in the normal IFN-γ protocol, there was a twofold increase in the proportion of Foxp3^+^ cells recovered (Fig. 3B), again highlighting the reciprocal relationship between IL-6 and Treg selection in this setting.

**Figure 4:**
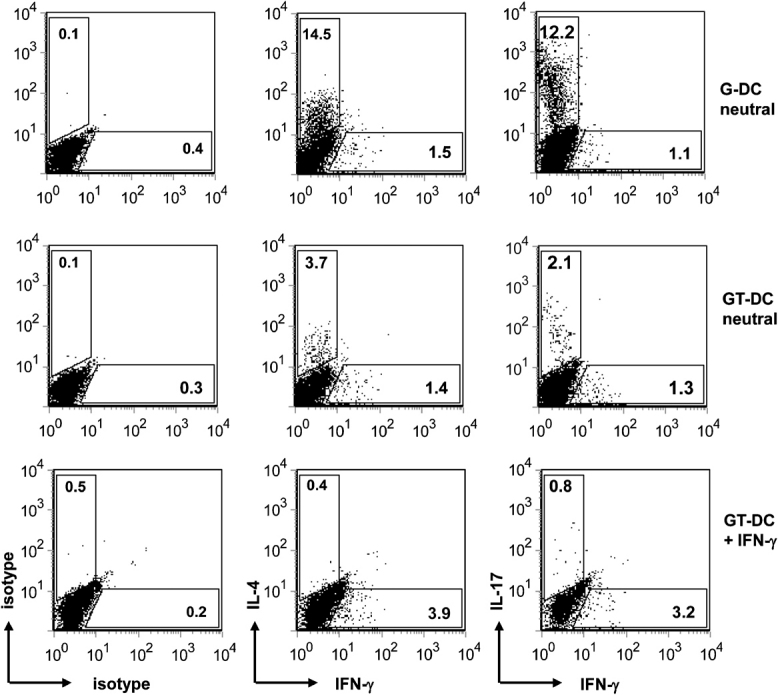
The *ex vivo* IFN-γ-conditioning protocol extinguishes Th2 and Th17 responses. Upper panel: Purified naive CBA CD4^+^ T cells were co-cultured with B10 GM-CSF differentiated BM DC (G-DC) without additional cytokines (neutral conditions). Middle panel: Purified naive CBA CD4^+^ T cells were co-cultured with B10 GM-CSF/TGF-β-differentiated BM DC (GT-DC) under neutral conditions. Lower panel: Purified naive CBA CD4^+^ T cells were co-cultured with B10 GM-CSF/TGF-β-differentiated BM DC in the presence of IFN-γ (5 ng/mL). Cells were restimulated under the same conditions on day 7, harvested on day 14, treated for 4 h with PMA and ionomycin, and stained for intracellular IFN-γ, IL-4 and IL-17 expression. Plots are gated on T cells and numbers in the dot plots indicate the frequency of cells in each region. Data are representative of two to three independent experiments.

The influence of TGF-β on IL-6 production in the APC population used in this conditioning protocol prompted us to examine the differentiation of Th17 cells, both under neutral conditions and in the presence of IFN-γ. As shown in Fig. 4, naive CBA CD4^+^ cells stimulated under neutral conditions with GM-CSF-conditioned DC make dominant Th2 and Th17 responses as judged by intracellular cytokine staining. However, when the same T-cell population is stimulated under identical conditions but with DC conditioned with GM-CSF+TGF-β these responses are reduced 4–6-fold, indicating that one important effect of TGF-β conditioning of the APC population is to skew the T-cell response away from both Th2 and Th17 pathways. It is interesting to note that, while the addition of IFN-γ has only a modest further effect on inhibition of Th17 responses, the production of IL-4 is reduced essentially to background levels. Thus, in addition to an enrichment of Foxp3^+^ Treg and the preferential elimination of Foxp3^–^ responders, stimulation of naive CD4^+^ T cells with TGF-β-conditioned APC in the presence of IFN-γ shapes the *ex vivo* T-cell response further by arresting the development of Th2 and Th17 cells, both of which have been implicated in destructive alloreactive responses.

### Endogenous TGF-β contributes to the emergence of Treg in this system

**Figure 5:**
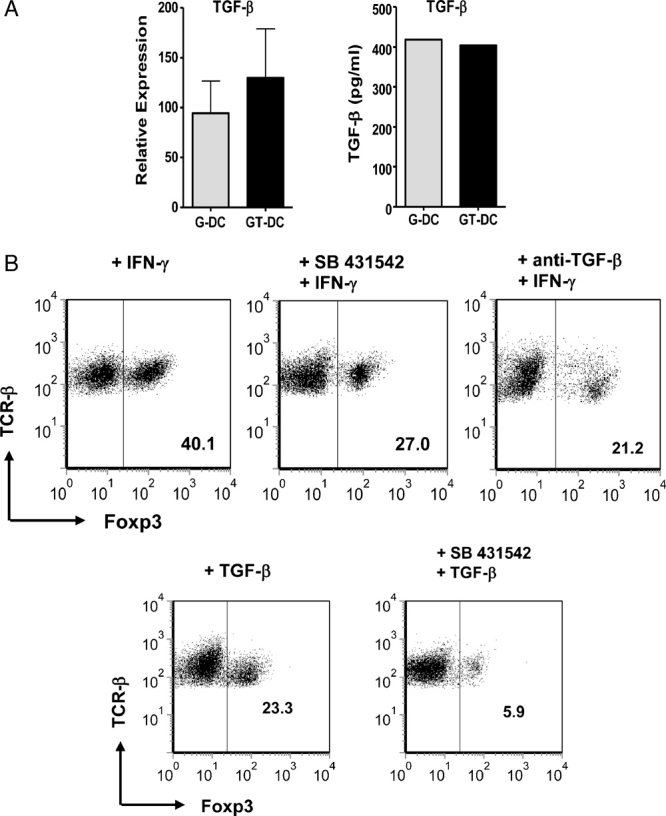
Enrichment of Foxp3^+^ cells depends on exogenous IFN-γ and endogenous TGF-β. (A) BM cells were cultured in the presence of 2 ng/mL GM-CSF with (GT-DC) or without (G-DC) 2 ng/mL TGF-β, harvested at day 6 and stimulated in the absence of T cells with recombinant CD40L for 24 h to mimic reverse stimulation by CD40–CD40L interactions. TGF-β RT-PCR data are normalized to the housekeeping gene GAPDH and are means±SD of two independent experiments (left panel). TGF-β production in supernatants was determined by ELISA and results were normalized to 4×10^5^ input cells in 2 mL medium (right panel). (B) Upper panel: Purified naive CBA CD4^+^ T cells were co-cultured with B10 GM-CSF/TGF-β-differentiated bone marrow DC in the presence of 5 ng/mL IFN-γ alone, or with IFN-γ+SB 431542 (10 mM), or anti-TGF-β antibody (10 mg/mL). Lower panel: Purified naive CBA CD4^+^ T cells were co-cultured with B10 GM-CSF/TGF-β-differentiated BM DC in the presence of TGF-β (2 ng/mL), without or with SB 431542 (10 mM). Cells were restimulated on day 7 under the same conditions, harvested on day 14, and intracellular Foxp3 expression was analyzed. Numbers in each dot plot indicate the frequency of cells in the quadrant. Data are representative of two independent experiments.

In view of the important role of TGF-β in other systems of Treg generation *ex vivo* 36–39, we asked whether the impact of TGF-β in this system is restricted to the effect of exogenous TGF-β in the APC-conditioning phase or whether TGF-β from endogenous sources is involved during the T-cell response itself. As shown in Fig. 5A, BM DC conditioned with GM-CSF alone express and secrete TGF-β when ligated with CD40L-Fc in a T-cell-free system and, importantly, this is unaffected by the addition of TGF-β itself. Thus, although exogenous TGF-β is not added during the second phase of the Treg generation protocol, the population of DC used to drive the T-cell response in the presence of IFN-γ has the capacity to produce endogenous TGF-β. In order to ask whether endogenous TGF-β plays a role in the emergence of Foxp3^+^ cells in the IFN-γ protocol, CBA CD4^+^ cells were co-cultured with GM-CSF/TGF-β B10 BM DC in the presence of IFN-γ (5 ng/mL), without or with neutralizing anti-TGF-β (10 μg/mL), or SB 431542 (10 μM), a selective inhibitor of activin receptor-like kinase 5, the TGF-β type I receptor 40,41. Cells were restimulated on day 7, harvested on day 14, and intracellular Foxp3 expression was analyzed. As shown in Fig. 5B, the proportion of Foxp3^+^ cells decreased from 40.1 to 27.0% when the TGF-β signalling inhibitor SB 431542 was added, and to 21.2% in the presence of anti-TGF-β antibody. The absolute number of Foxp3^+^ cells also decreased from 61.5±10.5×10^3^ in the standard IFN-γ conditioning to 13.5±2.5×10^3^ when SB 431542 was added (means±SD of two independent experiments). Although neither the inhibitor nor the antibody totally prevented an increase in the proportion of Foxp3^+^ cells, efficacy of this inhibitor concentration was confirmed by the fact that, when CD4^+^ cells were driven by APC in the presence of exogenous TGF-β alone, an increase in Foxp3^+^ cells was essentially abolished and the proportion remained virtually the same as in the starting population (Fig. 5B, lower panel). These data support previous reports that TGF-β signalling is important for the maintenance of Treg 36–39 and indicate that signalling by endogenous TGF-β also plays a role in this *ex vivo* IFN-γ-conditioning system. However, the TGF-β inhibitor data clearly show that the emergence of Foxp3^+^ cells in the IFN-γ protocol is the result if several interrelated factors of which endogenous TGF-β is an important but not predominant component.

### STAT1 signalling is essential for a Treg-dominant response

**Figure 6:**
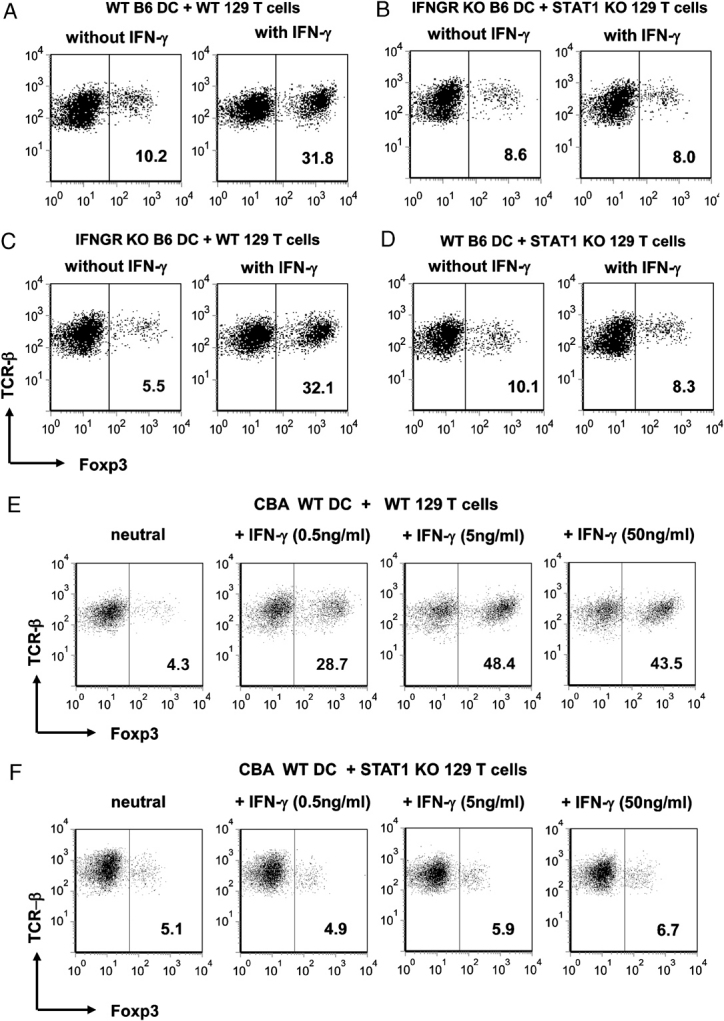
IFN-dependent selection of Foxp3^+^ cells is mediated *via* T-cell STAT1 signaling. (A) Purified naive wild-type (WT) 129 CD4^+^ T cells were co-cultured with wild-type B6 GM-CSF/TGF-β-differentiated BM DC in the absence or presence of IFN-γ (5 ng/mL). (B) Purified naive STAT1 knockout (KO) 129 CD4^+^ T cells were co-cultured with IFN-γ receptor knockout (IFNGR KO) B6 GM-CSF/TGF-β-differentiated BM DC in the absence or presence of IFN-γ (5 ng/mL). (C) Purified naive wild-type (WT) 129 CD4^+^ T cells were co-cultured with IFN-γ receptor knockout (IFNGR KO) B6 GM-CSF/TGF-β-differentiated BM DC in the absence or presence of IFN-γ (5 ng/mL). (D) Purified naive STAT1 knockout (KO) 129 CD4^+^ T cells were co-cultured with wild-type (WT) B6 GM-CSF/TGF-β-differentiated BM DC in the absence or presence of IFN-γ (5 ng/mL). Cells were restimulated on day 7, harvested on day 14, and intracellular Foxp3 expression was analyzed. Numbers in each dot plot indicate the frequency of cells in the quadrant; data are representative of two to three independent experiments. (E) Purified naive wild-type (WT) 129 CD4^+^ T cells were co-cultured with GM-CSF/TGF-β-differentiated CBA BM DC in the absence or presence of IFN-γ (0.5–50 ng/mL). (F) Purified naive STAT1 knockout (KO) 129 CD4^+^ T cells were co-cultured with GM-CSF/TGF-β-differentiated CBA BM DC in the absence or presence of IFN-γ (0.5–50 ng/mL). Cells were restimulated on day 7, harvested on day 14, and intracellular Foxp3 expression was analyzed.

CD4^+^ T cells and DC both express functional IFN-γ receptors and thus either population could be responding to IFN-γ in this *ex vivo* conditioning system. Since STAT1 phosphorylation is critical for IFN-γ signalling 42,43, we took advantage of IFN-γ receptor- (IFNGR KO) and Stat1-deficient (Stat1^−/−^) mice to determine in which population an IFN-γ response was essential. Naive wild-type 129Sv/Ev (129) or Stat1^−/−^ 129 CD4^+^ T cells were co-cultured with GM-CSF/TGF-β BM DC from either wild-type or IFNGR KO B6 mice. Although 129 and B6 mice are both H2^b^, these strains are mismatched for multiple minor histocompatibility antigens, demonstrated by the fact that 129 mice reject B6 skin grafts acutely (MST 16 days) and B6 APC drive vigorous proliferation of 129 CD4^+^ T cells *in vitro* as judged by CFSE dilution (64% of cells>1 division at day 7, data not shown). In order to identify the key responders to IFN-γ in this system, combinations were established where both populations can respond to IFN-γ (Fig. 6A, positive control), neither population can respond to IFN-γ (Fig. 6B, negative control), and where the IFN-γ response is restricted to either T cells or DC (Fig. 6C and D, respectively). Cells were restimulated on day 7, harvested on day 14, and intracellular Foxp3 expression was analyzed. As shown in Fig. 6A and C, the proportions of Foxp3^+^ cells were similar when CD4 T cells were driven by IFNGR KO B6 BM DC (32.1%) and when driven by wild-type B6 BM DC (31.8%), suggesting that a DC response to IFN-γ is not obligatory in this system. However, when Stat1^−/−^ 129 CD4^+^ T cells were co-cultured with DC from wild-type B6 mice, exogenous IFN-γ failed to increase the proportion of Foxp3^+^ cells recovered (8.3 *versus* 10.1%, with and without IFN-γ, respectively; Fig. 6D). In fact, the proportion of Foxp3^+^ cells was essentially the same as that seen when neither cell population was capable of responding to IFN-γ (Fig. 6B). Similar results were obtained using wild-type or Stat1^−/−^ 129 CD4^+^ T cells stimulated with GM-CSF/TGF-β BM DC from CBA (H2^k^) mice at each concentration of IFN-γ tested (Fig. 6E and F). Taken together, these results indicate that, in the IFN-γ-conditioning protocol described, T-cell signalling *via* STAT1 is essential for the enrichment of Foxp3^+^ T cells.

### Non-redundant role for STAT1 signalling in Th17 and Foxp3^+^ T-cell development

**Figure 7:**
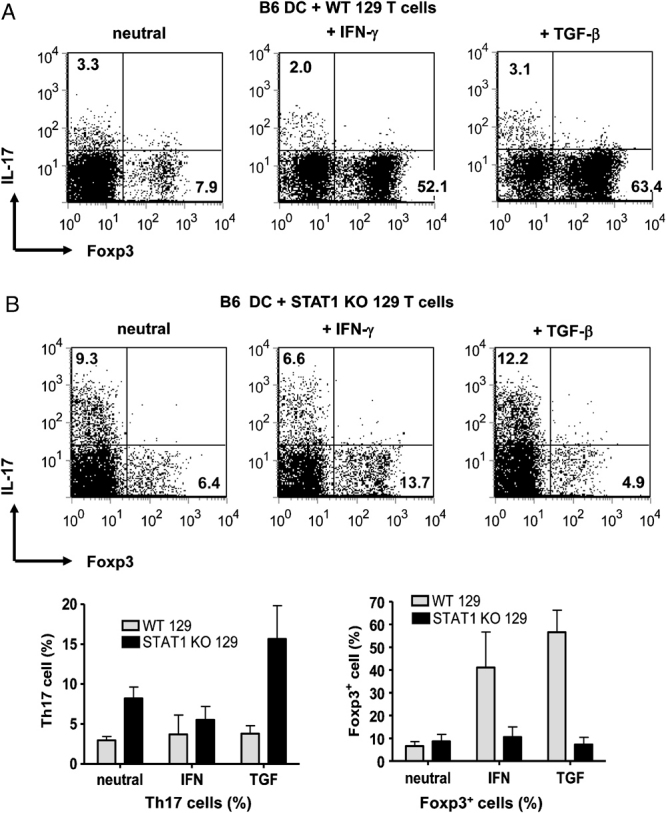
Non-redundant role for STAT1 signalling in the reciprocal development of regulatory and Th17 cells *ex vivo*. Purified naive CD4^+^ T cells from wild-type 129 mice (A) or STAT1 knockout 129 mice (B) were co-cultured with B6 GM-CSF/TGF-β-differentiated BM DC in the absence or presence of IFN-γ (5 ng/mL), or TGF-β (2 ng/mL). Cells were restimulated under the same conditions on day 7, harvested on day 14, treated for 4 h with PMA and ionomycin, and analyzed for intracellular Foxp3 and IL-17 expression in gated (TCR-β^+^) T cells. Figures in representative dot plots indicate the frequency of cells in each quadrant and summary histograms show means and SD of three independent experiments.

Cytokine signalling through STAT transcription factors is essential for T-cell differentiation. For instance, STAT4 and STAT6 are historically linked with Th1 and Th2 development, respectively, while more recently STAT3 has been linked with the development of Th17 cells and STAT5 with regulatory T cells 44. We wished to examine the impact of STAT1 deficiency on the emergence of Foxp3^+^ and Th17 T cells in the presence of IFN-γ, particularly since it has been shown that Th17 cell development is enhanced in Stat1^−/−^ mice 45. In the absence of exogenous cytokines (neutral conditions), Th17 cell development was enhanced in Stat1^−/−^ T cells (Fig. 7A *versus* B, left panels and summary histogram), consistent with Stat1-dependent regulation of Th17 responses in normal T cells 45, possibly by endogenous IFN-γ. The fact that addition of exogenous IFN-γ did not markedly inhibit Th17 responses suggests that, although the level of endogenous IFN-γ in these cultures is too low to drive the enrichment of Foxp3^+^ Treg (Figs. 1B and D and 7A), it is sufficient to influence Th17 cell programming (Fig. 7A, left and center panels). As with IFN-γ, the addition of TGF-β as the only cytokine increased the proportion of Foxp3^+^ cells 5–7-fold in wild-type cells (Fig. 7A, left *versus* right panel) but had no effect in Stat1^−/−^ cells (Fig. 7B, left *versus* right panels). These data indicate that STAT1 signalling plays an important role in Treg generation not only in the IFN-γ protocol but also in TGF-β-based protocols where T cells are driven by allogeneic APC. Indeed, STAT1 signalling may be a key factor in the balance between Treg and non-Treg populations in the wider context. In terms of absolute numbers, we have found that Stat1^−/−^ mice have essentially normal numbers of Foxp3^+^ cells, but these mice have profound splenomegaly and approximately ten times more CD4^+^ T cells with an activated phenotype (CD25^+^ Foxp3^−^) than wild-type controls (not shown).

### NO mediates the IFN-γ-conditioning-related Foxp3-dominant response

**Figure 8:**
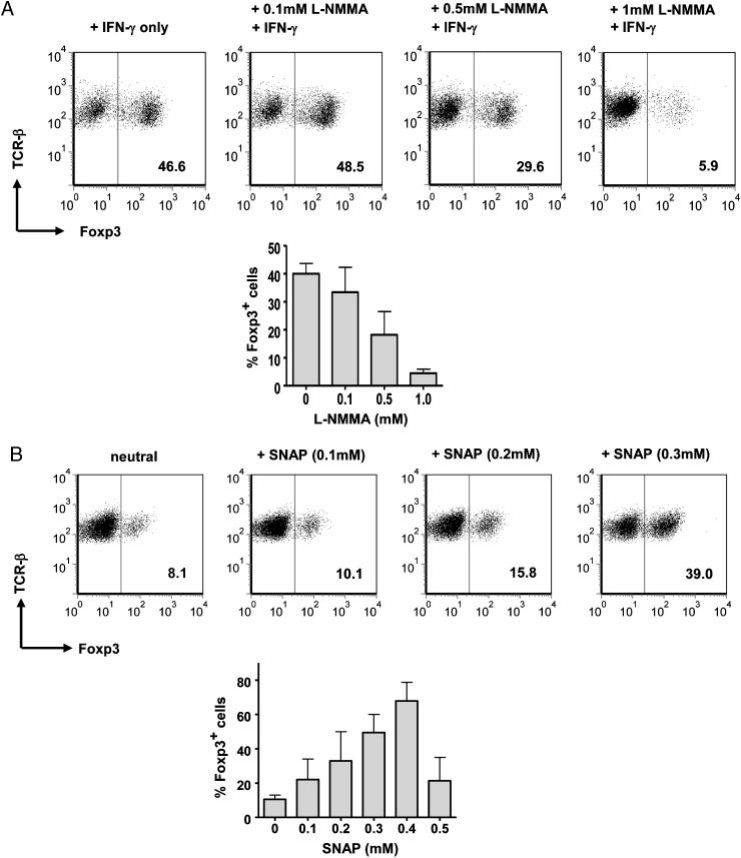
IFN-γ-conditioning results in NO-mediated Foxp3 upregulation. (A) Purified naive CBA CD4^+^ T cells were co-cultured with GM-CSF/TGF-β-differentiated B10 BM DC+5ng/mL IFN-γ in the absence or presence of the NOS inhibitor l-NMMA (0.1–1.0 mM). Cells were restimulated on day 7, harvested on day 14 and stained for TCR-β and intracellular Foxp3 expression. (B) Purified naive CBA CD4^+^ T cells were co-cultured with GM-CSF/TGF-β-differentiated B10 BM DC in the absence or presence of the NO donor SNAP (0.1–0.3 mM). No exogenous IFN-γ was added. Cells were restimulated on day 7, harvested on day 14 and stained for TCR-β and intracellular Foxp3 expression. Figures in representative dot plots indicate the frequency of Foxp3^+^ cells and histograms show means and SD of three independent experiments.

IFN-γ and STAT1 signals play an important role in the induction of NO 46,47 and real-time PCR analysis revealed a clear positive correlation between inducible NO synthase (iNOS) and Foxp3 expression in the *ex vivo* IFN-γ-conditioning system (data not shown). In order to ask whether NO is directly involved in the IFN-γ-conditioning protocol, naive CBA CD4^+^ T cells were co-cultured with GM-CSF/TGF-β B10 BM DC in the presence of IFN-γ (5 ng/mL), with or without *N*-methyl-l-arginine (l-NMMA) (0.1–1.0 mM), a widely used inhibitor of both constitutive and inducible forms of NOS 48,49. Cells were restimulated on day 7, harvested on day 14, and intracellular Foxp3 expression was analyzed. In the absence of l-NMMA, the proportion of Foxp3^+^ cells was 40.0±6.3%, but NOS inhibition resulted in a striking dose-dependent decrease: 33.4±15.4% with 0.1 mM, 18.2±14.4% with 0.5 mM and 4.5±2.6% with inhibitor at a final concentration of 1.0 mM (*p*<0.05, no inhibitor *versus* 1 mM inhibitor; Fig. 8A). In addition, the absolute numbers of Foxp3^+^ cells recovered also decreased from 30.0±8.5×10^3^ in the absence of l-NMMA to 13.5±2.1×10^3^ in the presence of 1 mM inhibitor. These data indicate that induced NO plays an essential role in the development of Foxp3^+^ Treg driven by IFN-γ conditioning.

We then asked whether a non-cellular source of NO could substitute for exogenous IFN-γ in the *ex vivo* conditioning protocol. Naive CBA CD4^+^ T cells were co-cultured with GM-CSF/TGF-β B10 BM DC in the absence of exogenous IFN-γ but with or without *S*-nitroso-*N*-acetyl-penicillamine (SNAP), a NO donor widely tested *ex vivo* 48,50,51. Cells were restimulated on day 7 under the same conditions, harvested on day 14, and intracellular Foxp3 expression was analyzed. As shown in Fig. 8B, the proportion of Foxp3^+^ cells increased in a concentration-dependent manner from 0.1 to 0.4 mM SNAP in that the proportion of Foxp3^+^ cells was 22.1±16.9, 33.0±24, 49.5±14.9 and 67.9±15.4% at 0.1, 0.2, 0.3 and 0.4 mM SNAP, respectively. Most striking is that the absolute number of Foxp3^+^ cells showed a clear concentration-dependent increase. The number of Foxp3^+^cells recovered was 25.0±2.8×10^3^, 35.5±2.1×10^3^ and 52.5±16.3×10^3^ at SNAP concentrations of 0.1, 0.2 and 0.3 mM, respectively. The yield decreased dramatically when SNAP was used at higher concentrations (0.4–0.5 mM), identifying a concentration of 0.3 mM as the optimal dose in this *ex vivo* system. Collectively, these data demonstrate that induced NO is an indispensable soluble factor mediating the development of Foxp3^+^ cells driven by alloantigen in the IFN-γ-conditioning protocol. Interestingly, at optimal concentrations of either exogenous IFN-γ or NO donor, the proportion of Foxp3^+^ cells was similar, but the provision of NO increased the yield of both total cells and Treg. This is partly explained by the fact that, as judged by annexin V staining, the level of cell death seen in the presence of the optimal concentration of NO donor (0.3 mM) is substantially lower than that seen in the presence of the optimal concentration of IFN-γ (7.9±4.0 and 16.6±8.0% for +SNAP and +IFN-γ, respectively; not shown), further reflecting the fact that exogenous IFN-γ in this system reduces the overall size of the responding T-cell pool due to its anti-proliferative and pro-apoptotic effects (Fig. 2 and 23–25,27,28). Indeed, an overall reduction in the size of the responding T-cell population may be an important prerequisite for regulation 52,53.

## Discussion

Treg cells generated or expanded *ex vivo* have significant potential as cellular therapeutics 54. Several such *ex vivo* protocols exist 37,38,55 and understanding the underlying mechanisms responsible for Treg generation will have an important influence in optimizing and identifying the most suitable protocols for clinical evaluation. In the current study, we have demonstrated that stimulation of mouse CD4^+^ T cells by allogeneic GM-CSF/TGF-β-conditioned immature DC in the presence of exogenous IFN-γ leads to a T-cell response dominated by the emergence of Foxp3^+^ regulatory T cells. Significantly, the resulting population can regulate rejection responses *in vivo* without further manipulation. Our working hypothesis at present is that regulation is mediated by Foxp3^+^ cells contained within the resulting population, but we cannot formally rule out a contribution by other regulatory populations such as IL-10-producing Tr1-like cells. However, as judged by intracellular cytokine staining, we have been unable to detect IL-10 production by IFN-γ-conditioned cells, and preliminary experiments conducted using other donor–recipient combinations have revealed a positive correlation between the degree of Foxp3 enrichment and regulation *in vivo*. We are currently conducting experiments in which sorted GFP^–^Foxp3^–^ cells will be used as the input population and resultant GFP^+^Foxp3^+^ and GFP^–^Foxp3^–^ cells will be assessed independently for functional regulatory capacity. We hope that this will allow us to determine definitively whether or not regulation is confined to alloantigen-driven Foxp3^+^ T cells.

The addition of exogenous IFN-γ appears to result in an overall selection for cells with a Treg phenotype *via* proliferation, conversion of non-Treg precursors and induction of cell death within the responding non-Treg population, but we have found little evidence for an increase in the absolute size of the Foxp3^+^ population compared with that recovered under identical conditions in the absence of IFN-γ. Despite this, the resulting cells regulate rejection in an alloantigen-specific manner and we speculate that alloantigen stimulation by GM-CSF/TGF-β BM DC in the presence of IFN-γ drives an overall shift in the specificity of Foxp3^+^ cells, resulting in a relatively oligoclonal population in much the same way as repeated *in vitro* stimulation selects for the survival of responding non-regulatory T cells in other settings. We are currently attempting to test this hypothesis by seeding TCR-transgenic T cells into polyclonal populations followed by alloantigen challenge in the presence of IFN-γ.

When total CD4^+^ T cells are used as the input population, there is clear evidence of preferential apoptosis or necrosis within non-Treg responders (Fig. 2D). The fact that IFN-γ can enhance apoptosis within activated T-cell populations has been known for many years 23–25, but the reasons why Treg appear to be less sensitive to IFN-γ-mediated cell death in this system are not known at present. One possibility is suggested by the fact that, in terms of class II and costimulatory molecule expression, BM DC conditioned with GM-CSF+TGF-β are much more heterogeneous than their classically derived counterparts and thus probably drive a similarly heterogeneous T-cell response resulting in fully activated Foxp3^–^ effector cells and those that have defaulted to express Foxp3^+^ because of suboptimal activation. The idea that suboptimal stimulation of responding T cells is a prerequisite for Treg generation in this system (as has been reported in the induction of Treg *in vivo* 56) is supported by the fact that, in our hands, IFN-γ failed to promote the conversion of Foxp3^–^/GFP^–^ CD4 cells to Foxp3/GFP^+^ cells driven by anti-CD3 mAb, a stimulus expected to provide supra-optimal T-cell activation (data not shown). Indeed, it was on this basis that GM-CSF/TGF-β-conditioned BM-derived DC were chosen as the preferred APC population in the IFN-γ-conditioning protocol because this pretreatment arrests DC maturation and retards MHC and costimulatory molecule upregulation. While we have not formally tested whether other isolated APC populations can also drive the selection of Foxp3^+^ Treg in the IFN-γ protocol, previous attempts using T-cell-depleted spleen cells as APC gave extremely unpredictable results. However, it seems likely that other immature APC would be capable of inducing the generation of Treg in this system, particularly if they retained the capacity for production of TGF-β (Fig. 5). It would be expected that suboptimally activated T cells would be less susceptible to AICD and, indeed, we have found, using CFSE-based assays, a clear correlation between T-cell proliferation and death in the IFN-γ conditioning protocol (data not shown). Furthermore, the fact that Treg tend to be anergic might also confer a relative resistance to AICD. An additional possibility is that alloantigen-driven Treg have an increased expression of anti-apoptotic molecules such as Bcl-x_l_, as has recently been reported in naturally occurring Foxp3^+^ Treg 57. We are currently investigating the expression of anti-apoptotic genes and IFN-γ receptors in Foxp3^–^ and Foxp3^+^ cells as a function of time in the IFN-γ-conditioning protocol.

Under neutral conditions, CD4^+^ T cells driven by allogeneic GM-CSF/TGF-β-differentiated DC tend to have an intrinsic bias toward Th2 and Th17 responses because these APC produce significant amounts of both TGF-β and IL-6. Significantly, this bias is prevented by the addition of exogenous IFN-γ without resulting in an overt Th1 response (Fig. 4), an observation entirely consistent with previous studies 58–60. Again, these data suggest that IFN-γ selectively inhibits development of the effector T-cell population while maintaining the development of Treg. Thus, the effect of exogenous IFN-γ in this system is to influence the development of a dominant Treg response by inducing death within Foxp3^–^ responders and by skewing the overall T-cell response away from Th2 and Th17 cells. In this context, it is interesting to note that, in a rather different *ex vivo* system where the emergence of Foxp3^+^ T cells is dependent on exogenous TGF-β 61, IL-4 appears to be more antagonistic than IFN-γ. This appears to be consistent with observations in the current study where one of the effects of exogenous IFN-γ is to inhibit Th2 development (Fig. 4).

Our data show that NO is critically involved in the *ex vivo* IFN-γ-conditioning protocol. IFN-γ is an efficient inducer of NOS activity 46, an observation consistent with the described effects of NO, an important effector molecule in immunity, particularly against intracellular pathogens. However, data are emerging indicating that NO also plays a regulatory role in immune responses 62. For example, iNOS-mutant mice develop significantly more pronounced Th1 responses than wild-type mice upon infection, and IFN-γ-induced NO can downregulate Bcl-2 expression and induce apoptosis of primed T cells 63,64. Williams *et al.* have shown that T cells can produce NO upon TCR signalling and that this is closely involved in AICD 65. In addition, NO can induce upregulation of IFN-γ receptor 2 expression on T cells, and these T cells are thus susceptible to IFN-γ 66. IFN-γ receptor signalling with subsequent STAT1 phosphorylation is critical for the function of IFN-γ 42,43. Stat1^−/−^ mice develop EAE and show enhanced generation of Th1 cells, suggesting that STAT1 signalling is not only non-obligatory for Th1 development but may play a negative feedback role on effector cell responses 67,68. A potential explanation for such observations has recently been provided by studies demonstrating that Stat1^−/−^ mice have enhanced Th17 responses 45, indicating that STAT1 signalling plays a critical negative role in Th17 cell differentiation. Indeed, both IFN-γ and IL-27 have been shown capable of suppressing Th17 development *via* signalling through STAT1 69 and a recent report has demonstrated that STAT1 phosphorylation can lead to the transcription of iNOS 47. Our data demonstrate that NO can play an important role as a downstream mediator of this effect since inhibition of NOS completely abrogated IFN-γ conditioning and provision of an NO donor in the absence of exogenous IFN-γ also resulted in a Foxp3^+^ Treg-dominant response.

The data presented in this study provide novel insights into the means and mechanism by which a dominant alloreactive regulatory T-cell population can be encouraged to develop *ex vivo*. The fact that the protocol results in partial selection of regulatory cells by shifting the balance between Treg and non-Treg populations suggests that, if used in concert, the IFN-γ protocol followed by polyclonal expansion might provide sufficient numbers of enriched regulatory cells for therapeutic use. Indeed, in this regard the preferential elimination of alloreactive non-Treg cells might confer a significant benefit. Recently, two other independent studies have demonstrated that exogenous IFN-γ can be used to influence Treg development *ex vivo*. In the first, Wang *et al.* demonstrated that polyclonal activation of mouse CD4^+^CD25^−^ T cells with anti-CD3 antibody in the presence of IFN-γ resulted in a population of CD25^+^ T cells that inhibited the development of EAE almost as effectively as naturally occurring Treg 70. In the second study, Brem-Exner *et al.* showed that exposure of lymph node cells *in vitro* to IFN-γ-conditioned monocytes led to an expansion of CD25^+^Foxp3^+^ T cells, and while the ability of these *ex vivo* generated/expanded cells to regulate responses *in vivo* was not formally tested, the fact that adoptive transfer of the IFN-γ-conditioned monocyte population led to a resolution of colitis in a mouse model of inflammatory bowel disease is consistent with an *in vivo* conversion driven by this APC population 8.

The results of the current study extend these observations significantly by demonstrating that IFN-γ can be used to shape the *ex vivo* T-cell response to alloantigens away from effector cell differentiation in favor of Treg development and that without further manipulation the resultant population can control acute allograft rejection. Although such approaches have potential therapeutic use in both transplantation and autoimmune disease, we believe that transplantation offers the unique advantage that APC from the graft donor can be used to drive Treg selection. Such antigen-specific activation is unlikely to be possible in autoimmunity, except in those cases where the antigens are well defined. Although living donor transplantation offers the most immediate possibility for donor-reactive Treg generation, the observation that some immunosuppressive agents are permissive and may select for regulatory T cells 55 suggests the possibility of combining Treg generation with short-course immunosuppression, to extend this type of approach to deceased donor transplantation.

## Materials and methods

### Mice

CBA.Ca (CBA, H2^k^), C57BL/10 (B10, H2^b^), C57BL/6 (B6, H2^b^), 129Sv/Ev (129, H2^bc^), CBA-recombination-activating gene 1 knockout (CBA-Rag^−/−^, H2^k^; kindly provided by Dr. D. Kioussis, Division of Molecular Immunology, National Institute for Medical Research, Mill Hill, London), IFN-γ receptor knockout B6 mice (IFNGR KO, H2^b^; kindly provided by Prof. Siamon Gordon, Sir William Dunn School of Pathology, Oxford, UK), and IL-6 knockout mice were obtained from and housed in the Biomedical Services Unit, John Radcliffe Hospital. Stat1^−/−^ 129 mice were purchased from Taconic Farm (Hudson, NY). *Foxp3gfp* knock-in (*Foxp3gfp*.KI) B6 mice and DBA/2 mice were bred and housed in the Transplant Research Center, Beth Israel Deaconess Medical Center, Harvard Medical School (Boston, MA) 33. Sex-matched mice between 6 and 12 wk of age at the time of first experimental procedure were used in all experiments.

### Reagents and mAb

The hybridomas TIB120 (anti-MHC class II) and RA3.6B2 (anti-B220) were obtained from the American Type Culture Collection; YTS169 (anti-CD8) and YTA3.1 (anti-CD4) 71 were kindly provided by Prof. H. Waldmann (Sir William Dunn School of Pathology, Oxford, UK). RM4-5-PerCP, 11B11-PE, XMG1.2-FITC and JES-19F1-PE were purchased from BD Pharmingen. The anti-Foxp3 antibody FJK-16s was obtained from eBioscience and used according to the manufacturer's instructions. l-NMMA and SNAP were purchased from Sigma-Aldrich.

### Cell purification

CD4^+^ T cells and CD25^−^CD4^+^ T cells were isolated using CD4 or CD25 MicroBeads (Miltenyi). Foxp3^–^ GFP^–^ CD4 T cells were flow-sorted (BD-Biosciences FACS-Vantage). On reanalysis, all populations were 95–99% pure.

### *In vitro* generation of BM DC

BM DC were generated from donor mice according to published methods 35,72. DC precursor-enriched BM cells were cultured with 2 ng/mL each of rmGM-CSF and rhTGF-β1 (PeproTech, London, UK). At day 6, DC were harvested, washed and counted prior to use.

### IFN-γ-conditioning protocol

Cell culture used RPMI 1640 containing 10% FCS, 2 mM l-glutamine, 0.5 mM 2-mercaptoethanol (Sigma) and 100 U/mL of penicillin and streptomycin (Sigma). Purified naive CD4^+^ T cells (5×10^5^ were co-cultured with 5×10^4^ allogeneic BM DC/2 mL well in RPMI 1640 medium containing 10% FCS in 24-well plates (Corning, NY), in the presence of 5 ng/mL exogenous rmIFN-γ (PeproTech). On day 7, half of the medium was replaced with fresh medium containing the same concentration of recombinant IFN-γ and the same number of DC. After two rounds of stimulation, cells were harvested for phenotypic analysis, or for functional evaluation in an adoptive transfer model.

### Adoptive transfer and skin transplantation

CBA-Rag^−/−^ mice were reconstituted intravenously with 1×10^5^ CD25^−^CD4^+^ cells from naive CBA with or without 2×10^5^ *ex vivo* conditioned cells. The following day, full-thickness B10 tail skin allografts were transplanted onto graft beds prepared on the left flank.

### Statistical analysis

Graft survival between transplant groups was compared using Kaplan–Meier survival curves and the Log-rank test (GraphPad Prism) with significance at *p*<0.05. Two-tailed comparisons were made using the Mann–Whitney test.

## Abbreviations

129: 129Sv/Ev
AICD: activation-induced cell death
B6: C57BL/6
B10: C57BL/10
BMDC: bone marrow-derived DC
CBA: CBA.Ca
CIA: collagen-induced arthritis
iNOS: inducible nitric oxide synthase
L–NMMA: N-methyl-l-arginine
MST: median survival time
NOS: nitric oxide synthase
SNAP: S-nitroso-N-acetyl-penicillamine

## References

[1] Weaver CT, Hatton RD, Mangan PR, Harrington LE (2007). IL-17 family cytokines and the expanding diversity of effector T cell lineages. Annu. Rev. Immunol..

[2] Ferber I, Brocke S, Taylor-Edwards C, Ridgway W, Dinisco C, Steinman L, Dalton D, Fathman C (1996). Mice with a disrupted IFN-γ gene are susceptible to the induction of experimental autoimmune encephalomyelitis (EAE). J. Immunol..

[3] Chu C-Q, Wittmer S, Dalton DK (2000). Failure to suppress the expansion of the activated CD4 T cell population in interferon γ-deficient mice leads to exacerbation of experimental autoimmune encephalomyelitis. J. Exp. Med..

[4] Willenborg DO, Fordham SA, Staykova MA, Ramshaw IA, Cowden WB (1999). IFN-γ is critical to the control of murine autoimmune encephalomyelitis and regulates both in the periphery and in the target tissue: a possible role for nitric oxide. J. Immunol..

[5] Manoury-Schwartz B, Chiocchia G, Bessis N, Abehsira-Amar O, Batteux F, Muller S, Huang S (1997). High susceptibility to collagen-induced arthritis in mice lacking IFN-γ receptors. J. Immunol..

[6] Vermeire K, Heremans H, Vandeputte M, Huang S, Billiau A, Matthys P (1997). Accelerated collagen-induced arthritis in IFN-γ receptor-deficient mice. J. Immunol..

[7] Ortmann RA, Shevach EM (2001). Susceptibility to collagen-induced arthritis: Cytokine-mediated regulation. Clin. Immunol..

[8] Brem-Exner BG, Sattler C, Hutchinson JA, Koehl GE, Kronenberg K, Farkas S, Inoue S (2008). Macrophages driven to a novel state of activation have anti-inflammatory properties in mice. J. Immunol..

[9] Markees TG, Phillips NE, Gordon EJ, Noelle RJ, Shultz LD, Mordes JP, Greiner DL, Rossini AA (1998). Long-term survival of skin allografts induced by donor splenocytes and anti-CD154 antibody in thymectomized mice requires CD4^+^ T cells, interferon-γ, and CTLA4. J. Clin. Invest..

[10] Fairchild RL (2003). The Yin and Yang of IFN-γ in allograft rejection. Am. J. Transplant..

[11] Wood KJ, Sawitzki B (2006). Interferon γ: a crucial role in the function of induced regulatory T cells *in vivo*. Trends Immunol..

[12] Le Moine A, Goldman M, Abramowicz D (2002). Multiple pathways to allograft rejection. Transplantation.

[13] Rocha PN, Plumb TJ, Crowley SD, Coffman TM (2003). Effector mechanisms in transplant rejection. Immunol. Rev..

[14] Saleem S, Konieczny BT, Lowry RP, Baddoura FK, Lakkis FG (1996). Acute rejection of vascularized heart allografts in the absence of IFN-γ. Transplantation.

[15] Bishop DK, Wood SC, Eichwald EJ, Orosz CG (2001). Immunobiology of allograft rejection in the absence of IFN-γ: CD8^+^ effector cells develop independently of CD4^+^ cells and CD40–CD40 ligand interactions. J. Immunol..

[16] Konieczny BT, Dai Z, Elwood ET, Saleem S, Linsley PS, Baddoura FK, Larsen CP (1998). IFN-γ is critical for long-term allograft survival induced by blocking the CD28 and CD40 ligand T cell costimulation pathways. J. Immunol..

[17] Guillonneau C, Hill M, Hubert F-X, Chiffoleau E, Herve C, Li X-L, Heslan M (2007). CD40Ig treatment results in allograft acceptance mediated by CD8^+^CD45RC^low^ T cells, IFN-γ, and indoleamine 2,3-dioxygenase. J. Clin. Invest..

[18] Boehm U, Klamp T, Groot M, Howard JC (1997). Cellular responses to interferon-γ. Annu. Rev. Immunol..

[19] O'Garra A (1998). Cytokines induce the development of functionally heterogeneous T helper cell subsets. Immunity.

[20] Gajewski T, Fitch F (1988). Anti-proliferative effect of IFN-γ in immune regulation. I. IFN-γ inhibits the proliferation of Th2 but not Th1 murine helper T lymphocyte clones. J. Immunol..

[21] Park H, Li Z, Yang XO, Chang SH, Nurieva R, Wang YH, Wang Y (2005). A distinct lineage of CD4 T cells regulates tissue inflammation by producing interleukin 17. Nat. Immunol..

[22] Harrington LE, Hatton RD, Mangan PR, Turner H, Murphy TL, Murphy KM, Weaver CT (2005). Interleukin 17-producing CD4^+^ effector T cells develop *via* a lineage distinct from the T helper type 1 and 2 lineages. Nat. Immunol..

[23] Liu Y, Janeway C (1990). Interferon γ plays a critical role in induced cell death of effector T cells: a possible third mechanism of self-tolerance. J. Exp. Med..

[24] Dalton DK, Haynes L, Chu C-Q, Swain SL, Wittmer S (2000). Interferon γ eliminates responding CD4 T cells during mycobacterial infection by inducing apoptosis of activated CD4 T cells. J. Exp. Med..

[25] Refaeli Y, Van Parijs L, Alexander SI, Abbas AK (2002). Interferon is required for activation-induced death of T lymphocytes. J. Exp. Med..

[26] Feuerer M, Eulenburg K, Loddenkemper C, Hamann A, Huehn J (2006). Self-limitation of Th1-mediated inflammation by IFN-γ. J. Immunol..

[27] Berner V, Liu H, Zhou Q, Alderson KL, Sun K, Weiss JM, Back TC (2007). IFN-γ mediates CD4^+^ T-cell loss and impairs secondary antitumor responses after successful initial immunotherapy. Nat. Med..

[28] Li X, McKinstry KK, Swain SL, Dalton DK (2007). IFN-γ acts directly on activated CD4^+^ T cells during mycobacterial infection to promote apoptosis by inducing components of the intracellular apoptosis machinery and by inducing extracellular proapoptotic signals. J. Immunol..

[29] Sawitzki B, Kingsley CI, Oliveira V, Karim M, Herber M, Wood KJ (2005). IFN-γ production by alloantigen-reactive regulatory T cells is important for their regulatory function *in vivo*. J. Exp. Med..

[30] Grohmann U, Orabona C, Fallarino F, Vacca C, Calcinaro F, Falorni A, Candeloro P (2002). CTLA-4-Ig regulates tryptophan catabolism *in vivo*. Nat. Immunol..

[31] Finger EB, Bluestone JA (2002). When ligand becomes receptor – tolerance *via* B7 signalling on DCs. Nat. Immunol..

[32] Mellor AL, Munn DH (2004). IDO expression by dendritic cells: tolerance and tryptophan catabolism. Nat. Rev. Immunol..

[33] Bettelli E, Carrier Y, Gao W, Korn T, Strom TB, Oukka M, Weiner HL, Kuchroo VK (2006). Reciprocal developmental pathways for the generation of pathogenic effector Th17 and regulatory T cells. Nature.

[34] Steinbrink K, Wolfl M, Jonuleit H, Knop J, Enk A (1997). Induction of tolerance by IL-10-treated dendritic cells. J. Immunol..

[35] Yamaguchi Y, Tsumura H, Miwa M, Inaba K (1997). Contrasting effects of TGF-β1 and TNF-α on the development of dendritic cells from progenitors in mouse bone marrow. Stem Cells.

[36] Marie JC, Letterio JJ, Gavin M, Rudensky AY (2005). TGF-β1 maintains suppressor function and Foxp3 expression in CD4^+^CD25^+^ regulatory T cells. J. Exp. Med..

[37] Taylor PA, Lees CJ, Blazar BR (2002). The infusion of *ex vivo* activated and expanded CD4^+^CD25^+^ immune regulatory cells inhibits graft-*versus*-host disease lethality. Blood.

[38] Zheng SG, Gray JD, Ohtsuka K, Yamagiwa S, Horwitz DA (2002). Generation *ex vivo* of TGF-β-producing regulatory T cells from CD4^+^CD25^−^ precursors. J. Immunol..

[39] Horwitz DA (2006). Transforming growth factor-β: taking control of T cells' life and death. Immunity.

[40] Inman GJ, Nicolas FJ, Callahan JF, Harling JD, Gaster LM, Reith AD, Laping NJ, Hill CS (2002). SB-431542 is a potent and specific inhibitor of transforming growth factor-β superfamily type I activin receptor-like kinase (ALK) receptors ALK4, ALK5, and ALK7. Mol. Pharmacol..

[41] Oida T, Xu L, Weiner HL, Kitani A, Strober W (2006). TGF-β-mediated suppression by CD4^+^CD25^+^ T cells is facilitated by CTLA-4 signalling. J. Immunol..

[42] Durbin JE, Hackenmiller R, Simon MC, Levy DE (1996). Targeted disruption of the mouse Stat1 gene results in compromised innate immunity to viral disease. Cell.

[43] Meraz MA, White JM, Sheehan KCF, Bach EA, Rodig SJ, Dighe AS, Kaplan DH (1996). Targeted disruption of the Stat1 gene in mice reveals unexpected physiologic specificity in the JAK-STAT signalling pathway. Cell.

[44] Yao Z, Kanno Y, Kerenyi M, Stephens G, Durant L, Watford WT, Laurence A (2007). Nonredundant roles for Stat5a/b in directly regulating Foxp3. Blood.

[45] Chen Y, Langrish CL, McKenzie B, Joyce-Shaikh B, Stumhofer JS, McClanahan T, Blumenschein W (2006). Anti-IL-23 therapy inhibits multiple inflammatory pathways and ameliorates autoimmune encephalomyelitis. J. Clin. Invest..

[46] Bronte V, Zanovello P (2005). Regulation of immune responses by l-arginine metabolism. Nat. Rev. Immunol..

[47] Robertson G, Hirst M, Bainbridge M, Bilenky M, Zhao Y, Zeng T, Euskirchen G (2007). Genome-wide profiles of STAT1 DNA association using chromatin immunoprecipitation and massively parallel sequencing. Nat. Methods.

[48] Koblish HK, Hunter CA, Wysocka M, Trinchieri G, Lee WMF (1998). Immune suppression by recombinant interleukin (rIL)-12 involves interferon γ induction of nitric oxide synthase 2 (iNOS) activity: inhibitors of NO generation reveal the extent of rIL-12 vaccine adjuvant effect. J. Exp. Med..

[49] Llovera M, Pearson JD, Moreno C, Riveros-Moreno V (2001). Impaired response to interferon-γ in activated macrophages due to tyrosine nitration of STAT1 by endogenous nitric oxide. Br. J. Pharmacol..

[50] Lander H, Sehajpal P, Levine D, Novogrodsky A (1993). Activation of human peripheral blood mononuclear cells by nitric oxide-generating compounds. J. Immunol..

[51] Huang FP, Niedbala W, Wei XQ, Xu D, Feng GJ, Robinson JH, Lam C, Liew FY (1998). Nitric oxide regulates Th1 cell development through the inhibition of IL-12 synthesis by macrophages. Eur. J. Immunol..

[52] Wells AD, Li XC, Li Y, Walsh MC, Zheng XX, Wu Z, Nunez G (1999). Requirement for T-cell apoptosis in the induction of peripheral transplantation tolerance. Nat. Med..

[53] Li Y, Li XC, Zheng XX, Wells AD, Turka LA, Strom TB (1999). Blocking both signal 1 and signal 2 of T-cell activation prevents apoptosis of alloreactive T cells and induction of peripheral allograft tolerance. Nat. Med..

[54] Wood K, Sakaguchi S (2003). Regulatory T cells in transplantation tolerance. Nat. Rev. Immunol..

[55] Battaglia M, Stabilini A, Roncarolo MG (2005). Rapamycin selectively expands CD4^+^CD25^+^FoxP3^+^ regulatory T cells. Blood.

[56] Kretschmer K, Apostolou I, Hawiger D, Khazaie K, Nussenzweig MC, von Boehmer H (2005). Inducing and expanding regulatory T cell populations by foreign antigen. Nat. Immunol..

[57] Minamimura K, Gao W, Maki T (2006). CD4^+^ regulatory T cells are spared from deletion by antilymphocyte serum, a polyclonal anti-T cell antibody. J. Immunol..

[58] Seder R, Paul W, Davis M, Fazekas de St. Groth B (1992). The presence of interleukin 4 during *in vitro* priming determines the lymphokine-producing potential of CD4^+^ T cells from T cell receptor transgenic mice. J. Exp. Med..

[59] Wenner C, Guler M, Macatonia S, O'Garra A, Murphy K (1996). Roles of IFN-γ and IFN-α in IL-12-induced T helper cell–1 development. J. Immunol..

[60] Bradley L, Dalton D, Croft M (1996). A direct role for IFN-γ in regulation of Th1 cell development. J. Immunol..

[61] Wei J, Duramad O, Perng OA, Reiner SL, Liu Y-J, Qin FX-F (2007). Antagonistic nature of T helper 1/2 developmental programs in opposing peripheral induction of Foxp3^+^ regulatory T cells. Proc. Natl. Acad. Sci. USA.

[62] Niedbala W, Cai B, Liu H, Pitman N, Chang L, Liew FY (2007). Nitric oxide induces CD4^+^CD25^+^ Foxp3^–^ regulatory T cells from CD4^+^CD25^−^ T cells *via* p53, IL-2, and OX40. Proc. Natl. Acad. Sci. USA.

[63] Wei XQ, Charles IG, Smith A, Ure J, Feng GJ, Huang FP, Xu D (1995). Altered immune responses in mice lacking inducible nitric oxide synthase. Nature.

[64] Tarrant TK, Silver PB, Wahlsten JL, Rizzo LV, Chan C-C, Wiggert B, Caspi RR (1999). Interleukin 12 protects from a T helper type 1-mediated autoimmune disease, experimental autoimmune uveitis, through a mechanism involving interferon γ, nitric oxide, and apoptosis. J. Exp. Med..

[65] Williams MS, Noguchi S, Henkart PA, Osawa Y (1998). Nitric oxide synthase plays a signalling role in TCR-triggered apoptotic death. J. Immunol..

[66] Allione A, Bernabei P, Bosticardo M, Ariotti S, Forni G, Novelli F (1999). Nitric oxide suppresses human T lymphocyte proliferation through IFN-γ-dependent and IFN-γ-independent induction of apoptosis. J. Immunol..

[67] Bettelli E, Sullivan B, Szabo SJ, Sobel RA, Glimcher LH, Kuchroo VK (2004). Loss of T-bet, but not STAT1, prevents the development of experimental autoimmune encephalomyelitis. J. Exp. Med..

[68] Nishibori T, Tanabe Y, Su L, David M (2004). Impaired development of CD4^+^CD25^+^ regulatory T cells in the absence of STAT1: increased susceptibility to autoimmune disease. J. Exp. Med..

[69] Batten M, Li J, Yi S, Kljavin NM, Danilenko DM, Lucas S, Lee J (2006). Interleukin 27 limits autoimmune encephalomyelitis by suppressing the development of interleukin 17-producing T cells. Nat. Immunol..

[70] Wang Z, Hong J, Sun W, Xu G, Li N, Chen X, Liu A (2006). Role of IFN-γ in induction of Foxp3 and conversion of CD4^+^CD25^−^ T cells to CD4^+^ Tregs. J. Clin. Invest..

[71] Qin S, Wise M, Cobbold S, Leong L, Kong Y, Parnes J, Waldmann H (1990). Induction of tolerance in peripheral T cells with monoclonal antibodies. Eur. J. Immunol..

[72] Inaba K, Inaba M, Romani N, Aya H, Deguchi M, Ikehara S, Muramatsu S, Steinman RM (1992). Generation of large numbers of dendritic cells from mouse bone marrow cultures supplemented with granulocyte/macrophage colony-stimulating factor. J. Exp. Med..

